# A pilot study of an online produce market combined with a fruit and vegetable prescription program for rural families

**DOI:** 10.1016/j.pmedr.2019.101035

**Published:** 2020-01-07

**Authors:** Christine M. Burrington, Thomas E. Hohensee, Nancy Tallman, Anne M. Gadomski

**Affiliations:** The Mary Imogene Bassett Hospital dba Bassett Medical Center, One Atwell Road, Cooperstown, NY 13326, USA

**Keywords:** Photovoice, Online produce market, Produce prescription, Produce consumption, Rural family, Food access

## Abstract

Despite being surrounded by farms, rural families are often at risk for diet-related diseases because of food disparities. Barriers such as distance and transportation to a full service grocery store, lack of cooking knowledge and skills, and the cost of fresh produce, force families to shop at convenience stores that carry predominantly unhealthy, processed foods. We combined a Fruit and Vegetable Prescription Program (F&VRx) with family cooking/nutrition classes and a pilot online produce shopping to promote lifestyle dietary changes in rural families. In 2 rural upstate New York communities school health staff referred low income families with one or more children at risk for chronic disease related to obesity for this pilot program. Each family was given a weekly online produce credit for 5 months, September/October 2017 through January/February 2018. Two monthly nutrition/cooking lessons were provided for the entire family. Evaluation was done using pre and post program surveys and Photovoice. Families took photos in response to the question “*How has the F&VRx program affected my family?”*. Redemption of online produce credit was 94% and class attendance was 80%. Fruit and vegetable consumption rose for children. Confidence, culinary skills, and food literacy increased slightly. Three months after program completion, 60% of the families continued weekly online produce shopping without the F&VRx. Healthy behaviors for shopping, preparing, and consuming fruits and vegetables can be increased using a F&VRx, online produce ordering, and family cooking classes.

## Highlights

• Rural families are at risk for diet-related diseases because of food disparities.
• An online produce market increases redemption of produce prescriptions.
• Photovoice illuminates the benefits and limits of produce prescription programs.
• Produce prescriptions, online produce market, and cooking lessons affect behavior.
• Fruit and vegetable consumption increases for families when they cook together.

## 1 Introduction/background

Rural families and their children are at risk for diet-related diseases such as diabetes, hypertension, and asthma due to many factors, including the lack of access to fresh fruits and vegetables (FV), the high cost of fresh produce, (Buyuktuncer et al., 2013) and lack of skills to prepare produce for consumption. This risk is compounded by the high availability of processed foods at gas stations or convenience stores. Rural communities are often considered ‘food deserts’ defined by the US Department of Agriculture (USDA) as “having to travel more than 10 miles to a full service grocery store”. In addition, poor food literacy and lack of transportation contribute to this problem.

Rural communities are usually poverty-dense and food insecure. Nationally, 1 in 8 households is food insecure (12.3%) and a majority of them have children (Gundersen and Ziliak, 2015). Adult FV consumption of 5 servings per day in rural upstate New York is 33.5% for fruits and 20.3% for vegetables (NYS, BRFSS). Relief of rural hunger and increasing access to healthy options has been attempted by introducing the Supplemental Nutrition Assistance Program (SNAP), school backpack programs for children, and food pantries. In this pilot program study, we evaluated another strategy: a Fruit and Vegetable Prescription Program (F&VRx) coupled with an online produce market and family cooking/nutrition classes.

By giving healthcare providers the means to provide patients with “prescriptions” for fresh produce for redemption at local farmers’ markets, grocery store chains, and independent markets, F&VRx have positively affected health outcomes (Wholesome Wave) such as lowering HbA1c and improving maternal and neonatal health (Trapl et al., 2016, Bryce et al., 2017). However, in rural areas, farmers’ markets are seasonal and full service grocery stores are out of reach.

Improving access to FV is not in itself the solution to improving health outcomes. Changes in knowledge, attitude and practice must accompany the ‘prescription’ to sustain the lifestyle change. Because fresh food is not easily accessible to rural families, meals need advanced planning. Limited trips to a full service grocery store necessitates that some perishable foods such as FV cannot be purchased too far in advance. Skills to prepare meals served at a future time have to be learned in order to reduce waste and add diversity to the family meal.

The objectives of this pilot study were to evaluate whether a strategy using F&VRx, cooking/nutrition classes and an online produce market would 1) increase FV consumption, 2) increase the variety of FV in the family’s meal patterns, 3) change attitudes towards consuming FV, 4) increase nutrition and culinary knowledge to strengthen this healthy behavior, and 5) sustain change in lifestyle behaviors after the F&VRx ends.

## 2 Methods

### 2.1 Setting and participants

Two small, rural communities in upstate New York were chosen based on a history of engagement in wellness initiatives with schools, worksites and community groups; they also both have School Based Health (SBH) centers. SBH and the school nurse in each school district were asked to recommend 5 low-income families that had one or more children at risk for chronic disease associated with obesity (BMI at or above the 85th percentile) and who also qualified for Free and Reduced-Price School Meals (family income < $46,435 for a family of four). A letter, containing a description of the program, expectations of the participants, and incentives for completion, was sent to each family, inviting their participation. A follow-up phone call confirmed their interest and ability to participate.

With healthcare providers identifying eligible families, we partnered with a community organization, Pathfinder Village (PV), to provide the produce. PV is a residential community for individuals with Down syndrome and other developmental disabilities. PV provides on-campus job training and community internships. The organization created a weekly produce market called Pathfinder Produce to give retail training to their campus residents and to provide fresh produce at cost for surrounding communities. PV added an *online* produce market in order to increase FV access only for families enrolled in our pilot study.

We also partnered with Cornell Cooperative Extension (CCE) to offer classes with developed, tested lessons to teach meal planning, food literacy, and cooking skills to promote the use of FV.

Eleven families were enrolled in September through October 2017. One family withdrew for personal reasons in December 2017. The five families that enrolled later in October received FV for the full 5 months but missed 2–4 CCE nutrition/cooking classes. Family size ranged from 1 to 5 children, ages 9 months to 20 years old. All members of the family were invited to the cooking classes. The parent who did grocery shopping for the family completed the survey which was administered each month. The number of families participating was limited by the available space for teaching and cooking.

### 2.2 Intervention

At the initial class, F&VRx and Photovoice, a community-based participatory research method, were described in greater detail. Signed, informed consents for the program and for photos were obtained and a baseline survey was administered. Written and oral instructions for the online produce market were given. Each family received an assortment of spices, kitchen utensils, and culinary information sheets.

The F&VRx was implemented over 5 months, a period of time shown in previous studies to change behavior (Bryce et al., 2017, Alcazar et al., 2017). A weekly online credit of $15 for a family of three, $20 for four, and $25 for five or more was given to each family. These amounts were approximated based on a Georgia study (Wholesome Wave, Georgia FVRx, 2017). Families logged onto the Pathfinder Produce site, viewed the FV available for that week, and placed their order anytime between Tuesday at noon and Wednesday at midnight. Produce was delivered to the PV campus every Thursday morning, then orders were delivered to a local site for pick up by families on Thursday afternoon. Weekly purchases of FV were tracked by dollar amount and by item for each family using online data monitoring. If they neglected to place an order, the credit was lost for that week. If the produce they received was damaged or unacceptable, they were given a credit for that amount for the following week.

Community sites in each village provided a venue for produce delivery for family pick up and for CCE to conduct classes. The intervention selected for the study was a modified version of CCE’s evidence-based Expanded Food and Nutrition Education Program (EFNEP), which “influences nutrition and physical activity behaviors of low-income families, particularly those with young children.” (Cornell Cooperative Extension of Otsego and Schoharie Counties) Completion was defined as redeeming 80% of the F&VRx ‘vouchers’ (online credit) and attending 70% of the classes. Incentives for completion of the study were a slow cooker and a cookbook. CCE monitored attendance by requiring the parent(s) to sign in.

### 2.3 Survey

To evaluate the F&VRx intervention, the same survey was administered monthly to identify patterns of shopping for FV, the consumption of FV by both the parent and children and to ascertain confidence levels with regard to cooking and trying new recipes. The first survey and the last survey given to each family were used for pre and post survey results. Surveys were completed by the parent responsible for grocery shopping. Parents were asked a range of daily servings of FV consumed for each child and themselves (None, 1–2, 3–4, 5 or more), but did not include if they purchased FV used in class.

Four questions were asked about cooking confidence using a 10 point scale.

How confident do you feel about:

a. cooking for your family with basic ingredients?
b. tasting food that you have not eaten before?
c. preparing and cooking new foods and recipes?
d. following a simple recipe?

Families were asked to rate the following two statements to assess household food insecurity (Often true, Sometimes true, Never true, I don’t know).

In the last 30 days:

a. The food that we bought just didn’t last and we didn’t have money to buy more food.
b. We cut the size of our meals or skipped meals because there wasn’t enough money for food.

### 2.4 Photovoice

Participants were introduced to Photovoice (Wang and Burris, 1997) that uses participant photos to document life conditions as *they* see them. Participants were asked to photograph answers to the question: “How has the Fruit and Vegetable Prescription program affected my family?”. Disposable cameras were offered but all participants chose to use personal cell phones. Participants were instructed to adhere to three rules when taking photos: (1) Respect the rights and privacy of others; do not take pictures of people without their knowledge, (2) avoid taking a picture if it harms you or the subject of your photo and (3) take pictures relevant to the question and appropriate for children to view.

At the end of the program, families were asked to submit 1–6 photos by email. Photos submitted by each family were uploaded into a presentation for viewing by the group. The families gathered for a meal provided by the program and were asked to describe each of their photos using the SHOWeD method as a guide (Shaffer, 1983). Their answers were audio recorded. The following questions were asked: “Who (or what) is this a photo of”, “Where is it taken”, “What is going on in this picture”, and “How does it answer the question**:** *How has the F&VRx program affected my family?*”. After all the photos for one family were displayed and discussed, the following questions were asked: “How have your meals changed”, “What new cooking techniques have you learned”, and “What new ways have you learned to do food budgeting”.

The discussion continued with the entire group by asking the following questions:

a. What did you think about ordering produce online?
b. If this were an ideal world, what would be the next best step for you and the community to help your family continue to eat more fruits and vegetables?
c. How should your community change to increase access to fruits and vegetables?
d. (Children) What did you like best about this program?

The recordings were transcribed and analyzed by two researchers who did open coding independently to generate codes pertaining to motivators and barriers to eating FV. A thematic analysis was done to group the codes under four broad themes (Thomas and Harden, 2008).

All methods and procedures were approved by the Mary Imogene Bassett Hospital Institutional Review Board (IRB).

### 2.5 Sustainability

Three months after the intervention ended, PV was contacted to provide a report indicating how many families continued to use the market without a prescription and an average dollar amount spent.

## 3 Results

### 3.1 Attendance and voucher redemption rate

Three of the 10 families attended all 10 classes, four attended 8 or 9 classes and 2 attended 6 classes. One family registered late and attended 4 of the 6 available to them, resulting in an overall attendance rate of 80%. The range of voucher redemption by families was 69–100% with a mean of 94%.

### 3.2 Purchasing patterns

In reviewing purchase patterns, most families ordered produce suggested by recipes provided in the culinary classes. Seasonal availability of produce prevented the study from demonstrating a change in variety of FV based on choice.

### 3.3 Survey data

**Fig. 1:**
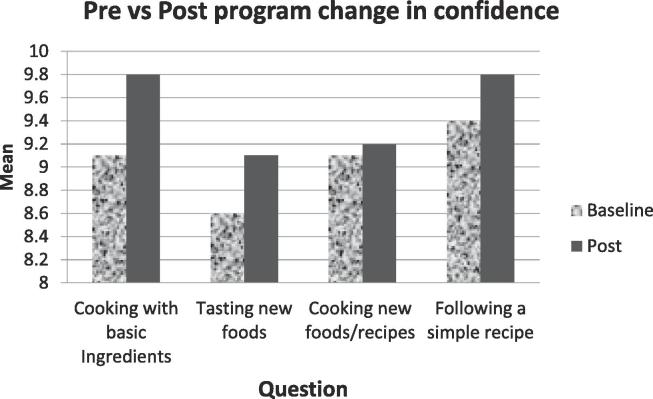
Parents showed a slight increase in confidence about their cooking skills and attitudes towards trying new foods.

**Fig. 2:**
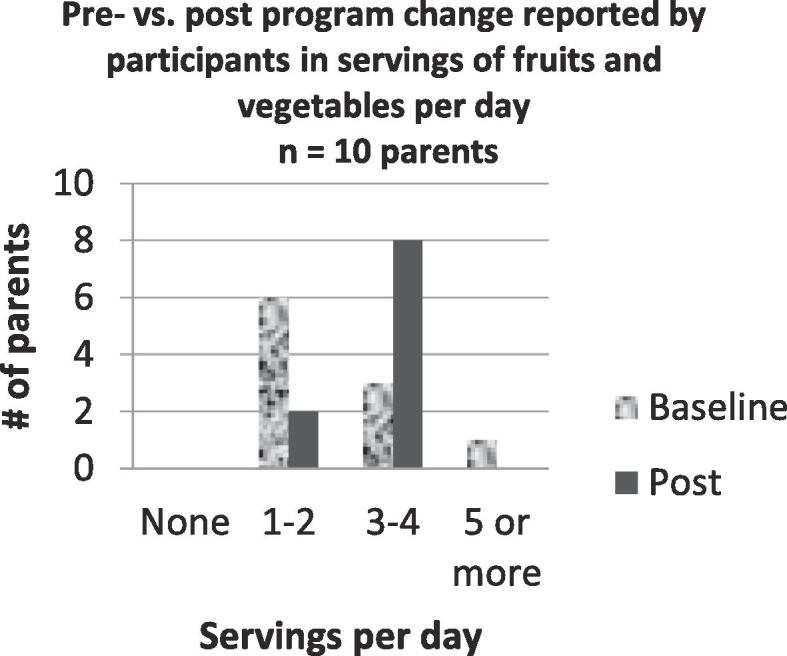
Parents reported consuming more FV, approaching the recommended 5 or more servings per day.

**Fig. 3:**
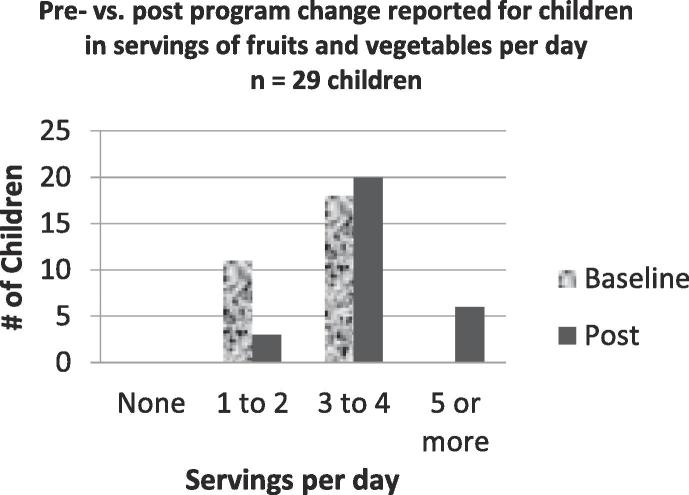
Parents reported an increase in FV servings per child per day.

**Fig. 4:**
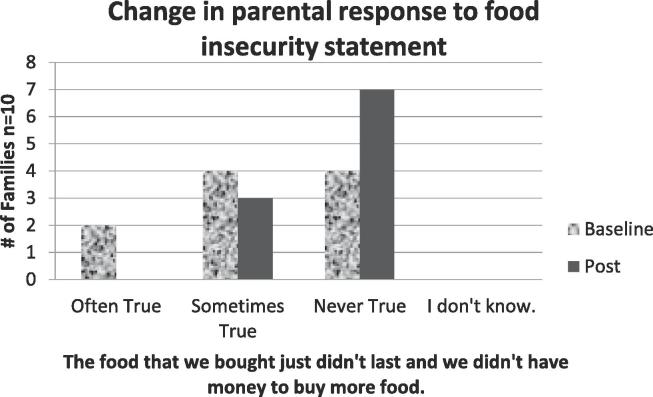
Six families indicated financial constraints at the start of the program, compared to three at program completion.

Survey results demonstrated increased confidence for cooking with basic ingredients, tasting new foods, cooking new recipes with FV, and following a simple recipe (Fig. 1). Questions about parents’ consumption of FV showed little change (Fig. 2**)**. However, parents reported that children’s consumption showed a trend towards eating five or more servings per day (Fig. 3). Following the intervention, more families reported that it was never true that the food they bought didn’t last the full month (Fig. 4).

### 3.4 Photovoice: results documenting themes 1 through 4

**Fig. 5:**
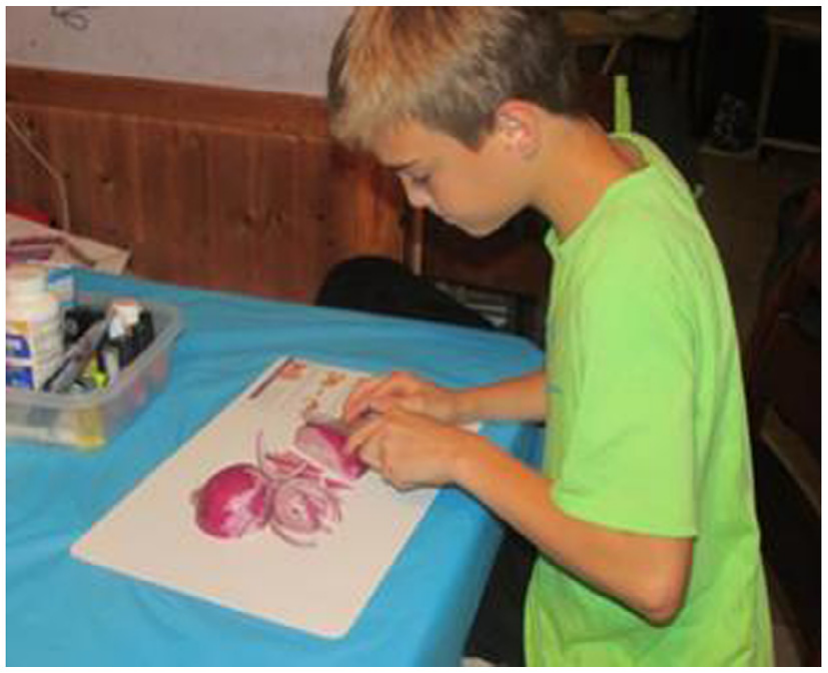
“That would be my 12 year old, son at home, who has a bad habit of cutting himself with knives. … And he has gotten better at class, using them right, helping cook dinner that night.”

**Fig. 6:**
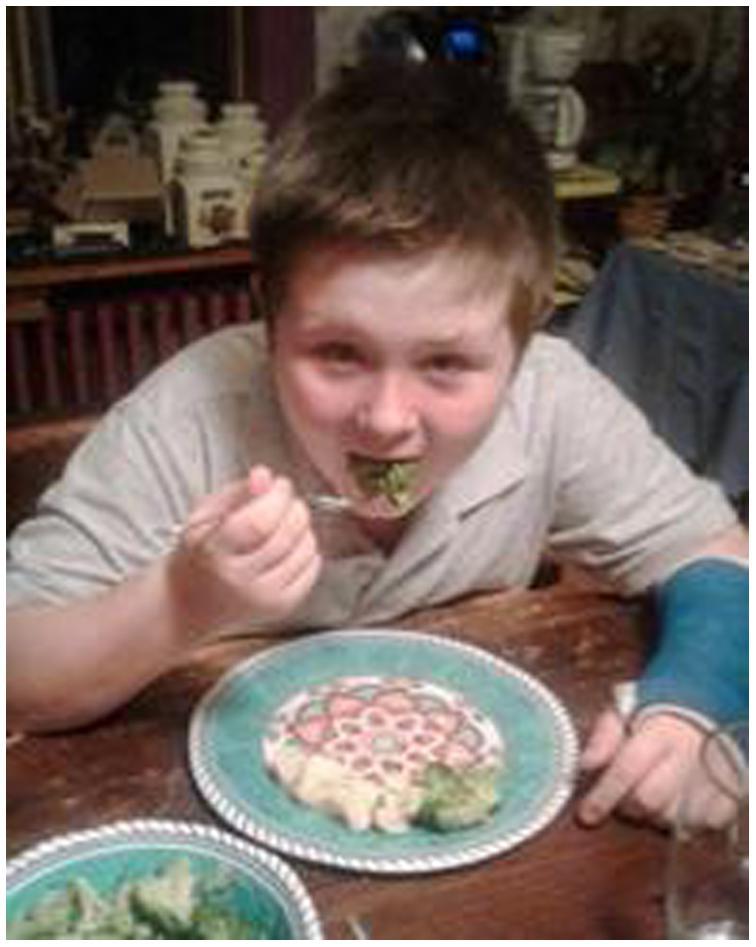
the children are more apt to eat vegetables now….. and fruits; they’re seeing different ways that you can prepare them that are healthy. And so they are more into trying different things, as opposed to not liking something. They are more willing to try it.“

**Fig. 7:**
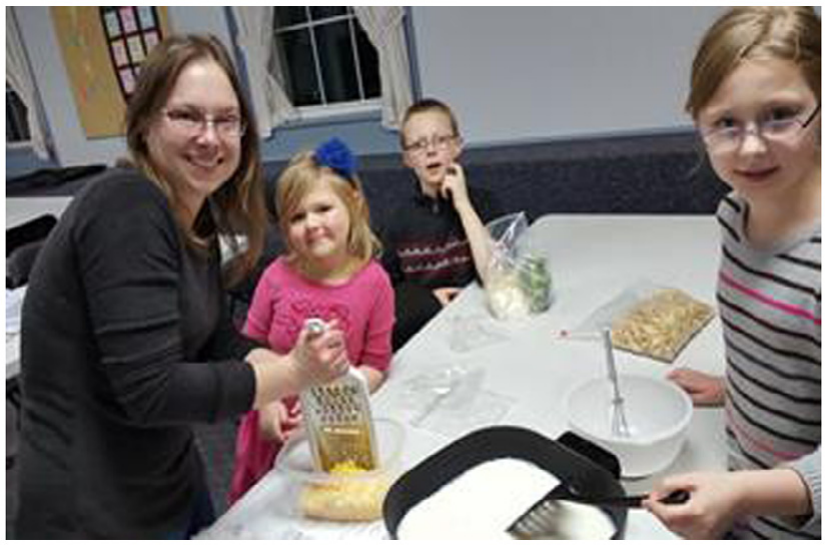
plus we’re preparing more (meals) together versus just grabbing something quick”.

**Fig. 8:**
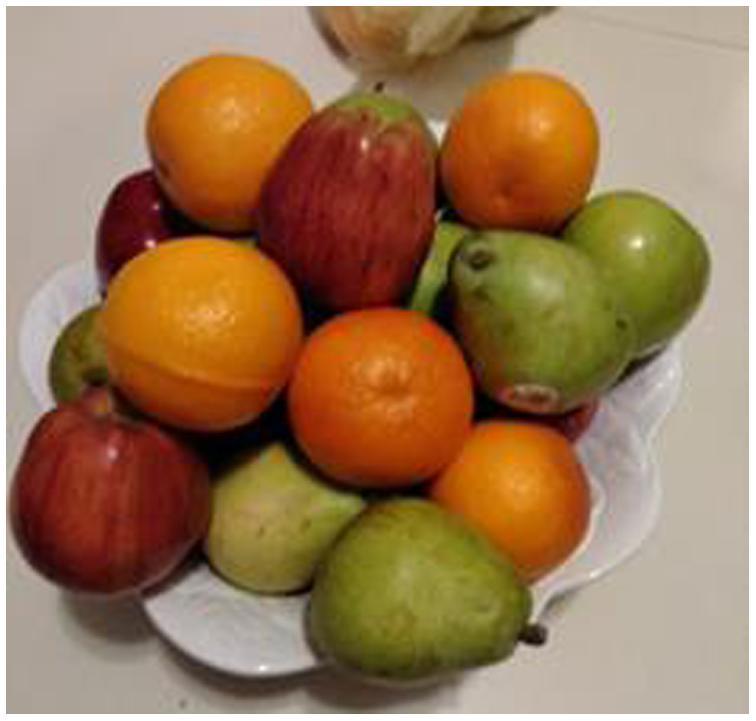
we started doing this (F&VRx) and then I’ve got all this fruit and I’m like, ‘Where am I gonna put it?’ So I cleaned out the bowl and it sits on the counter and now the kids just walk by and grab a fruit and keep walking …. instead of looking for cookies or chips…!”

Four main themes emerged from discussions of the photos and are summarized in Table 1, Table 2, Table 3, Table 4. Each of these themes is discussed further in the following paragraphs. Fig. 5, Fig. 6, Fig. 7, Fig. 8 include photographs participants took to illustrate each theme.

**Table 1:** Description of theme 1 with relevant quotes.

Theme 1	Quotes:
**Cooking classes enable families to efficiently plan and prepare FV for meals.** - Preserving FV for future use - Minimizing waste - Choosing FV when ripe - Planning meals before shopping - Knife skills - Easier to cook at home with more hands - Recipe experimentation - Using existing recipes - Cutting the FV differently	When we have extra grapes and they don't get eaten,… we throw them in the freezer (to use for smoothies). I learned how a pear is ripe and when it's not. I never knew that before. So you can buy them hard and then…… they don't rot. Different ways of cutting veggies…. being more precise to get it done faster. Making sure I have a list when I go (shopping) and definitely being more prepared. Like having a menu, definitely has been huge! Just teaching (the children) how to be more involved in and actually letting them cook instead of doing it myself. ….So I think finding/making the time to let them come in and help me, instead of….kicking them out of the kitchen. Well, I think our biggest take-away is what we're using; how we're using it. …like the Brussel sprouts in salads instead of just cooking them. ….vegetables instead of pasta.

**Table 2:** Description of theme 2 with relevant quotes.

Theme 2	Quotes:
**Consumption of FV increases because of change in attitude.** – Taking pride in preparation of food helped children overcome dislike of certain foods. – Knife skills gave more confidence to children to cook *and* to parents to let them help.	He’s attempting to eat a beet he helped prepare…that night. So he was ready to give it a try….and decided he liked them!

**Table 3:** Description of theme 3 with relevant quotes.

Theme 3	Quotes:
**Cooking at home increases family time** - Cooking together - Cooking a recipe they learned in class	Me and the kiddos were working together. We were, I forget what we were making that night, but grating cheese and there was just a job that everybody could do …. everyone got involved. (in my picture) we're making the Black Bean Soup.

**Table 4:** Description of theme 4 with relevant quotes.

Theme 4	Quote:
**Accessibility to FV alters food choices.** - Having FV every week through ease of online shopping - Fruit in a bowl is easier to grab and eat	You go to the store and just look at it and it's like “I ain't got time!“ Here (at the computer/home) you got time to actually,…… think about what you want ahead of time so you know you're not rushed at a grocery store when a 3 year old's with you.

*Theme 1: Cooking classes enable families to efficiently plan and prepare FV for meals.*

All participants agreed that the nutrition/cooking classes were essential to their using the FV made accessible by the program. Next to eating the food they prepared, children remarked that using a knife to chop vegetables was the most enjoyable activity of the class. The increased confidence in prepping veggies gained by the children reduced the concerns that parents had to allow them in the kitchen to help. Parents also remarked that *they* learned how to cut vegetables more efficiently with a knife which saved time.

New shopping habits such as meal planning, buying FV in season, and reviewing store brochures for bargains before going to the grocery store were adopted by the parent who did the food shopping. The classes also taught how to choose produce either to eat immediately or later in the week. Simple food preservation tips helped participants minimize waste.

Traditional recipes, such as macaroni and cheese, were modified to be made more nutritious by learning how to make the cheese sauce with reduced fat and by adding vegetables such as broccoli. Smoothies containing added spinach were introduced in the class and readily adopted at home. Adding FV to salads to make them more colorful was a new concept to participants. With the availability of free produce parents felt the freedom to experiment by adding vegetables to home recipes.

*Theme 2: Consumption of FV increases because of change in attitude.*

Some children in the study expressed a dislike for certain vegetables like broccoli and cauliflower. However, helping to prepare them for the family gave them the courage to “try” them. Other studies have shown that children are more likely to eat or at least try to eat food they have prepared (Berge et al., 2016, Van der Horst et al., 2014). Mastering knife skills made some vegetables more ‘approachable’ for consumption by children *and* parents.

*Theme 3: Cooking at home increases family time.*

Photovoice conversations revealed that recipes practiced in class were being tried at home and that the children were helping. With the added help it was more fun to cook a tastier meal together.

*Theme 4: Accessibility to FV alters food choices.*

Each of the families remarked that having a weekly produce order led them to use more FV in their meal plans. The free produce allowed families to experiment with new vegetables or new ways to use vegetables in home recipes while not compromising the family food budget. Cost is a factor in their choice of processed foods over FV. One parent noted, “You can buy a processed burger for a dollar or a salad for $8, which is difficult.”

Prior to the intervention, a fruit bowl was not part of one family’s home life. With fruit now available, the mother cleaned out a bowl containing ‘junk’ and replaced it with fruit, placing it on the counter for her children to grab and go. This small change made it easier for all family members to choose fruits.

The online produce market made it easier for parents to shop. After viewing the availability of FV online, planning meals was more thoughtful and considered more achievable.

### 3.5 Sustainability

Three months after the intervention ended, 6 of the 10 families continued to use the market either online or in person spending $15–20 per week without the prescription.

## 4 Discussion

The objectives of the F&VRx were to increase consumption and variety of FV in family meals, change attitudes towards consuming FV, and increase nutrition and culinary practices to strengthen and sustain these desired outcomes. Survey results and Photovoice evaluation demonstrated that this pilot program coupled with nutrition/cooking classes and an online produce market increased consumption of FV for children, and provided the skills and increased food literacy to help parents incorporate FV in meal planning and preparation. Most parents felt confident about preparing a meal and following a simple recipe at the start of the program. Learning about food preservation and how vegetables can be added to commonly used recipes enabled parents to improve nutrition and to make better use of their food dollars.

Despite the program taking place during the winter months, class attendance was surprisingly high at 80% likely due to two reasons. There are few educational opportunities in rural areas in which whole families can participate *and* the classes provided a special time for families to interact by cooking together.

Responses to the food insecurity questions showed that we were targeting families that would benefit from this program. Household food insecurity is a strong predictor of higher healthcare utilization and increased healthcare costs (Tarasuk et al., 2015). It is one social determinant of health that when addressed, has a profound impact on health outcomes.

Nationally, other studies have shown the positive impacts of F&VRx on participants. For example, Wholesome Wave, a national program that makes fresh FV affordable and accessible to communities, reported that of over 13,000 individuals from both rural and urban areas receiving FV prescriptions, 69% increased FV consumption (Rural Health Information Hub). In our study, only the children increased their consumption of FV as reported by parents. This is consistent with other studies that demonstrate young children consume more FV when they help prepare family meals (Van der Horst et al., 2014, Quelly, 2019). Other studies have shown that involving adolescents in food preparation improves their dietary quality and changes their eating patterns (Berge et al., 2016). Increased self-efficacy in cooking skills in young adults also predicts better nutrition a decade later (Utter et al., 2018).

Our redemption rate of 94% is higher than the 70% documented in other studies (Anderson et al., 2001, Trapl et al., 2016). This higher rate may be attributed to the online credit given to families eliminating the potential loss or forgetting of vouchers when picking up produce. Weekly reminders and allowing 36 h in which to place an order online also increased convenience for busy parents.

Photovoice images (Fig. 5, Fig. 6, Fig. 7, Fig. 8) and stories of the families brought to light the benefits and limitations of the program that survey questions did not capture. This qualitative evaluation technique highlighted the importance of online ordering and the impact of the cooking lessons to promote FV consumption.

One strength of this pilot program was that the cooking classes, which engaged the whole family, were offered during the dinner hour when families could eat what they had just prepared in the classes. The weekly online credit and produce market provided by PV enabled high participation and produce redemption rates as well as continued use after the program ended. An unanticipated benefit from this program was that increasing cooking skills for older children allowed them to get involved in the kitchen and thus reduce the overall time needed to prepare a meal. This finding is supported by a study (Santarossa et al., 2015) that found teaching young adolescents practical cooking skills led to more healthful diets and increased family dinner frequency.

One limitation of this study was the small sample size. Only 10 families were enrolled due to limited funding and venue space for the classes. However, caretakers varied in terms of age and/or being married or single.

Another limitation is that participants had to rely on PV employees to select FV they ordered. Some complained, for example, that certain produce was too ripe. PV replaced any unsatisfactory produce with a credit for the following week. Seasonality of produce over the duration of the intervention prevented any determination of change in variety. And finally, the Photovoice protocol calls for teaching participants some basic photo lessons which we did not provide. Though all adult participants had cell phones to take pictures, the quality of the photos provided was poor in some cases.

## 5 Conclusion

The objective of this pilot program to increase the consumption of FV in 10 rural low-income families was achieved by removing some common barriers: access to fresh produce, the skills and knowledge to prepare them for consumption, and the cost of FV. The program was enhanced by coupling it with cooking/nutrition lessons and online produce shopping. As a result of this pilot study, PV has expanded the F&VRx with online produce shopping by introducing a mobile market truck to reach families and individuals on Medicaid in Otsego County. They will study the effects of F&VRx with online purchasing and home delivery on hospitalization rates of children and adults with chronic disease related to obesity.
